# Medical Items

**Published:** 1897-03

**Authors:** 


### *MEDICAL ITEMS.*

---

DOCTOR, don't forget the Georgia Medical Association in Macon, April 20th, 21st, and 22d.

---

DR. DAVID CERNA, of Galveston, has been appointed by the Mexican Government consul at that port.

---

THE Trustees of the New York Polyclinic and Hospital (lately destroyed by fire) have decided to rebuild on the site of their former building. Work will be begun immediately.

---

DR. JOHN B. HAMILTON, of Chicago, will deliver the address on State Medicine at the next meeting of the American Medical Association, in the place of Dr. Jerome Cochran, deceased.

---

THE Maryland *Medical Journal* says that "during the past ten years over 600 papers have been written on the tonsils." This function of the tonsil—to be written on—has not hitherto been mentioned.

THE PASSING OF THE CIGARETTE.—The Lower House of the Tennessee Legislature has passed unanimously a bill prohibiting the sale of cigarettes in that State. If the bill becomes a law it will go into effect May 1, 1897.

DOCTOR—Just place this thermometer under your tongue, Mrs. Peque, and keep your lips closed tightly. Mr. Henry Peque (after a few minutes of speechless delight)—What will you take for that instrument, Doc.?—*Puck.*

MEDICAL EDUCATION FIFTY YEARS AGO.—In an old medical journal (May, 1847) we find the statement: "There are now *thirty-seven* medical colleges in the United States, and we may add, a *few more of the same sort* still in contemplation."

DR. HENRY L. BATTLE, of Wadley, Ga., died February 15th, in the seventy-first year of his age. He was one of the original members of the Georgia Medical Association (1849). Only two of the charter members of the Association now survive.

THE *Laryngoscope*, published in St. Louis, has been selected as the official organ for the year 1897, of the Laryngological Section of the New York Academy of Medicine. This selection, and the great probability of the same journal being chosen by other Laryngological, Rhinological and Otological societies as their official organ, would indicate that *The Laryngoscope* has become what its proprietors stated they intended to make it, *i. e.*, the American Journal of Record for the specialties represented.

THE Tennessee Centennial and International Exposition, at Nashville, will open May 1st and continue six months. There will be a Section on Medical History and Literature, and separate space will be provided for medical, surgical, and sanitary appliances. This department will be supplied with medical journals, and efforts will be made to make it specially interesting to visiting physicians. This Section will be in charge of Dr. Deering J. Roberts, of Nashville (chairman); Dr. G. C. Savage and Dr. S. S. Crocket, of Nashville; Dr. E. S. Miller, of Johnson City; Dr. D. E. Nelson, of Chattanooga; Dr. I. A. McSwain, of Paris, and Dr. T. J. Happel, of Trenton.

---

"DOCTOR" FRED RUTLAND, of Chicago, the proprietor of the diploma mill known as the Wisconsin Eclectic Medical College, has been arrested for using the mails for fraudulent purposes. He and his college have been furnishing diplomas and conferring degrees (any old degree) at prices ranging from twenty-five to fifty dollars. It is said that W. C. Offutt, of Savannah, Ga., had received five distinct degrees between June 26th and September 25th of last year. It is estimated that Rutland has conferred about 900 degrees and received about $30,000 from his "College" since last May.—*Daily Papers.*

---

A REPORT adopted at the second Pan-American Congress, held last November at Mexico City, has been presented to the United States Senate regarding a Department of Public Health for the United States. The bill proposes gathering information from every possible source relating to public health, sanitation, food, and diseases. It asks the appointment by the President of a statistic and compiling secretary, at a salary of $8,000, with an assistant at $5,000. The information thus gathered is to be published for issuance each week. The committee, in addition, recommends the passage of uniform laws, National and State, regulating the importation, exportation, sale, and inspection of food, laws regarding the sale of drugs and chemicals, sanitation of railroad cars, hospitals, and jails, and an appropriation of money by the States and General Government for the scientific investigation of public health matters in this and foreign countries.—*Medical News.*

